# The quality of Internet information relating to 2019-nCov transmission control in dental practice

**DOI:** 10.4317/jced.57573

**Published:** 2021-03-01

**Authors:** Fabio Camacho-Alonso, José Lacal-Luján

**Affiliations:** 1 DDS, PhD. Department of Oral Surgery, University of Murcia, Murcia, Spain

## Abstract

**Background:**

To date, the quality of the Internet information regarding the control and management of 2019-nCov virus transmission in dental clinics has not been evaluated. The aim of this study was to evaluate the quality of Internet information about the control of 2019-nCov transmission in dental practice.

**Material and Methods:**

Internet websites were identified daily using two search engines: Google and Yahoo! during the week from 20-06-2020 to 26-06-2020, applying the search term “2019-nCov transmission control in dental practice.” The first 100 consecutive sites identified in each search were visited and classified. The quality of information contained in each website was analyzed using the Journal of the American Medical Association (JAMA) benchmarks, whether the website had been granted the Health on the Net Foundation Code of Conduct (HONcode), and a new tool for evaluating the quality of Internet websites providing information relating to 2019-nCov transmission control in dental practice, which awards a score of 0-40 points (8-13: poor; 14-26: medium; and 27-40 high).

**Results:**

After the exclusion of duplicates, non-functioning websites, books/journals, irrelevant websites, or websites not in English, a total of 30 websites were evaluated. Only 6.66% fulfilled all four JAMA benchmarks, none had been granted the HONcode, and only 10% presented high quality information.

**Conclusions:**

The quality of Internet information about 2019-nCov transmission control in dental practice is poor. This study points to the need to improve the quality of information available on the Internet relating to 2019-nCov transmission control in dental practice.

** Key words:**2019-nCov, COVID-19, transmission control in dental practice, Internet, quality of information.

## Introduction

On December 31st 2019, The Wuhan Municipal Health Commission (Hubei province, China) reported a group of 27 cases (including seven severe cases) of a pneumonia of unknown etiology. Patients shared common exposure at a wholesale market in the city of Wuhan that sells diverse foodstuffs such as fish and seafood, but also live animals. On January 7th 2020, the Chinese healthcare authorities identified the agent causing the outbreak, a new type of virus belonging to the Coronaviridae family, later named as 2019-nCoV. On March 11th 2020, the rapid worldwide transmission of the virus caused the World Health Organization (WHO) to declare this a global pandemic (1). Corona viruses (enveloped, single stranded, positive direction RNA virus family, in which electron micrographs of spherical particles create an image reminiscent of the solar corona) are a family of viruses that causes infections in human beings (such as zoonotic disease) and a wide variety of animals. The corona viruses that affect humans (HCoV) may produce clinical manifestations that range from slight respiratory symptoms (as in the case of HCoV-229E, HCoV-NC63, HCoV-HKU1, and HCoV43) (2), to more intense or even lethal symptoms such as those produced by Severe Acute Respiratory Syndrome (SARS-CoV-1, which broke out in 2002 and 2003 with more than 8,000 contagions in seven countries, and a 10% death rate), or Middle East Respiratory Syndrome (MERS-CoV, with 2,500 reported cases since 2012 mainly in Saudi Arabia with a death rate of 34%) (3).

Due to the similarity between 2019-nCoV and SARS-CoV-1, it has now been renamed as SARS-CoV-2 by the Coronaviridae Study Group (CSG) of the International Committee on Taxonomy of Viruses (ICTV). The spike glycroproteins of SARS-CoV-1 and SARS-CoV-2 share 76% identity at the amino acid level. In the same way, both viruses are able to enter human cells via the angiotensin 1 converting enzyme 2 (ACE2) receptor, expressed on type II alveolar cells (AT2) of lung (4) and other tissues (kidney proximal tubule cells, myocardial cells, bladder urothelial cells, upper esophagus, enterocytes from colon and ileum, cholangiocytes, bladder urothelial cells, and epithelial cells of oral mucosa). Nevertheless, biophysical assays indicate that SARS-CoV-2 binds its common receptor ACE2, with a 10-20-fold higher affinity than SARS-CoV-1 (5). This new virus, 2019-nCoV or SARS-CoV-2 causes a novel pneumonia that the WHO denominated Corona Virus Disease 19 (COVID-19) on 11th February 2020 (6). In this new disease, after an incubation period of 1-14 days, the infected individual may remain asymptomatic or develop some of the more common functional symptoms (fever over 38ºC, dry cough, shortness of breath or dyspnea, fatigue, myalgia and/or arthralgia, and headache) or other less common symptoms (dizziness, nausea, vomiting, diarrhea, abdominal pain, anosmia, rhinorrhea, dysgeusia) (7) or dermatological signs such as erythematous rash, urticaria, or vesicle formation (8). The disease can cause death when irreversible alveolar damage is produced after progressive respiratory failure (9).

The most frequent transmission route between individuals (from saliva, blood or other body fluids) for 2019-nCoV and SARS-CoV-2 is direct inhaling via the airway (oral or nasal mucosa) though coughing, sneezing, droplet inhalation (respiratory droplets with a diameter of between 5 and 10 µm or droplet nuclei with a diameter less than 5 µm) making correct protection against aerosols formed during medical procedures essential for the protection of healthcare staff (10). Saliva-to-eye contagion has also been shown to be another transmission mechanism, with or without ocular signs such as conjunctival hyperemia, chemosis, epiphora, or increased ocular secretions; this makes eye coverings important for protecting healthcare staff (11). Oro-fecal (12) and sexual transmission (13) are also possible. Another important transmission route that must be considered for the protection of healthcare staff is indirect transmission via contaminated working surfaces. Although the virus’s survival depends on temperature and humidity conditions (mean temperature and humidity allowing indirect transmission being 5-11ºC and 47-79% respectively, while 4ºC and 80% humidity are the optimal conditions for transmission) (14), SARS-CoV-2 can remain on surfaces for at least 30 minutes without losing infectivity (15).

By the middle of April 2020, there were 83,400 confirmed infections with 3,349 fatalities in China, and 1,623,873 confirmed cases with 99,617 deaths in 209 other countries (16,17). In this situation, new and effective protocols for preventing 2019-nCov transmission control in dental practice were urgently needed. To this end, Peng *et al.* (18) published an article in April 2020 recommending a protocol for controlling 2019-nCov transmission in dental practice based on all the information about the topic published to date in the literature. These recommendations were divided into eight different sections: patient evaluation, hand hygiene, personal protective measurements for dental professionals, mouthrinse before dental procedures, rubber dam isolation and high-volume suction, anti-retraction high-speed dental handpiece, disinfection of clinic settings, and management of medical waste.

Given the current global situation, information relating to 2019-nCov transmission control in dental practice is of great clinical importance. It is known that 90% of adults use the Internet to obtain information, and 53% of dental healthcare professionals use the Internet to update information relating to 2019-nCov transmission control in dental practice. To date, several info-epidemiological studies have assessed healthcare information about COVID-19 prevention without applying any quality scoring systems (19), while others have assessed online information in this context using some scoring system or quality evaluation tool, such as the Health on the Net Foundation Code of Conduct (HONcode), the Journal of the American Medical Association (JAMA) benchmarks, or the DISCERN instrument (20).

But to date, the quality of the Internet information regarding the control and management of virus transmission in dental clinics has not been evaluated. For this reason, the aim of this study was to evaluate the quality of information provided by Internet websites relating to 2019-nCov transmission control in dental practice.

## Material and Methods

-Search Strategy

Internet websites were identified using two search engines: Google and Yahoo!. During the week 20-04-2020 to 26-04-2020, the two search engines were used at the same hour each day (11:00 a.m.) entering the search term “2019-nCov transmission control in dental practice.” The first 100 consecutive sites in each search were visited and classified. The search was not restricted in terms of file format or domain. The following were excluded: websites in a language other than English; duplicate sites; non-functioning websites; websites requiring a password to enter; book reviews or journal abstracts; and websites that did not contain information relevant to “2019-nCov transmission control in dental practice.” As the study investigated published information, no ethical approval was required. The quality of the online information collected was assessed two independent observers with wide experience in evaluating the quality of online information in dentistry (21-23). Any disagreements were resolved by consensus prior to the final analysis. 

The websites were categorized in terms of: affiliation (commercial, non-profit organization, university of medical center, or government); specialization (exclusively related to “2019-nCov transmission control in dental practice”, or a more extensive site containing an area dedicated to “2019-nCov transmission control in dental practice”); and the type of content (medical facts, clinical trials, questions and answers, or human interest stories).

Two well-known and validated tools - JAMA benchmarks and HONcod – were used to evaluate the quality of the information found relating to 2019-nCov transmission control in dental practice. In addition, a new tool was also used based on the study by Peng *et al.* (18).

JAMA benchmarks

The JAMA benchmarks (24) evaluate a series of criteria: authorship (authors and contributions, their affiliations, and relevant credentials should be displayed); attribution (clear references and sources of all content should be provided); disclosure (ownership of the website, sponsorship, advertising, underwriting, commercial funding or support sources, and any potential conflicts of interest); and currency (dates of initial posting and updating of the content should be noted). For each criterion (authorship, attribution, disclosure, and currency) the website receives 1 point generating a final score of 0-4 points.

HONcode

The presence or absence of the HONcode issued by the Health on the Net Foundation was recorded. This HONcode is a code of conduct for medical and healthcare websites that ensures the reliability and usefulness of the online information provided, indicated by a distinctive logo posted on the website. The HONcode is awarded when the website fulfils an 8-point code of conduct consisting of: authority, complementarity, confidentiality, attribution, justifiability, transparency of authorship, transparency of sponsorship, and honesty in advertising and editorial policy (25).

New tool for evaluating the quality of online information relating to 2019-nCov transmission control in dental practice based on study by Peng *et al.* (18) 

In April 2020, Peng *et al.* (18) published an article recommending a protocol for 2019-nCov transmission control in dental practice, based on all the information published to date in the literature. These recommendations were divided into eight domains: patient evaluation, hand hygiene, personal protective measurements for dental professionals, mouthrinse before dental procedures, rubber dam isolation and high-volume suction, anti-retraction high-speed dental handpiece, disinfection of the clinic settings, and management of medical waste. Based on these items, we developed a new tool for evaluating the quality of internet information sites providing information relating to 2019-nCov transmission control in dental practice. A score of 1-5 was awarded for each item as it appeared on the website. Scores were awarded according to the following criteria.

1. Patient evaluation: 1 point, no information included; 2 points, a contact-free forehead thermometer recommended; 3 points, three enquiries made relating to presence of fever, respiratory problems, and possible contacts with infected people (“Do you have fever or have you experienced fever within the past 14 days?” “Have you experienced a recent onset of respiratory problems such as a cough or difficulty in breathing within the past 14 days?” “Have you come into contact with a patient with confirmed 2019-nCoV infection within the past 14 days?”); 4 points, presence of information about the outcomes of 2 and 3-point scores; 5 points, presence of information about the outcomes of 2 and 3-point scores together with accurate information on patient management (management options: a. if a patient replies “yes” to any of the screening questions, and his/her body temperature is below 37.3ºC, the dentist should defer the treatment until 14 days after the exposure event; b. if a patient replies “yes” to any of the screening questions, and his/her body temperature is above 37.3ºC, the patient should be quarantined immediately; c. if a patient replies “no” to all the screening questions, and his/her body temperature is below 37ºC, the dentist can treat the patient; d. if a patient replies “no” to all the screening questions, but his/her body temperature is above 37ºC, the patient should be referred for medical care).

2. Hand hygiene was scored as: 1 point, no information available; 2 points, hand hygiene measures used before and after one or two of a list of five procedures (patient examination, dental procedures, touching the patient, touching the surroundings and equipment without disinfection, and touching the oral mucosa/damaged skin or wound/blood/bloody fluid/secretion/ or excreta); 3 points, hand hygiene measures used before and after three of the five procedures; 4 points, hand hygiene measures used before and after four of the five procedures; 5 points, hand hygiene measures used before and after all five of the five procedures.

3. Personal protective measurements for dental professionals was scored as: 1 point, no information available; 2 points, primary protective measurements are recommended (standard protection for staff in clinical settings: wearing disposable working cap, disposable surgical mask, working clothes, using protective goggles or face shield, and disposable latex or nitrile gloves); 3 points, secondary protective measurements are recommended (advanced protection for dental professionals: wearing disposable doctor’s cap, disposable surgical mask, protective goggles, face shield, and working clothes with disposable isolation clothing or surgical clothes outside and disposable latex or nitrile gloves); 4 points, tertiary protection with strengthened protection using Personal Protective Equipment (PPE); 5 points, the information for a 4-point score is available together with the recommendation to restrict clinical practice to dental emergencies only.

4. Mouthrinse before dental procedures was scored as: 1 point, no information; 2 points, mouthrinse use before dental procedures is recommended, stipulating chlorhexidine; 3 points, 1% hydrogen peroxide or 0.2% povidone recommended (one or the other but not both); 4 points, both 1% hydrogen peroxide and 0.2% povidone are recommended; 5 points, information for 4 points present with additional information about the action of oxidative agents on 2019-nCov.

5. Rubber dam isolation and high-volume suction was scored as: 1 point, no information available; 2 points, high volume suction recommended; 3 points, rubber dam isolation recommended; 4 points, both 2- and 3-point score information included; 5 points, 2- and 3-point score information included accompanied by recommendation of other manual devices to minimize aerosol generation such as CarisolvTM (MediTeam, Goteborg, Sweden) and hand scaler for caries removal, or manual root scaling and planing without the use of periodontal burs.

6. Anti-retraction high-speed dental handpiece was scored as: 1 point, no information available; 2 points, anti-retraction high-speed dental handpiece with some anti-reflux features recommended, but no specific system stipulated; 3 points, anti-retraction high-speed dental handpiece is recommended stipulating specific approved systems (such as anti-retraction valves); 4 points, both 2- and 3-point information present; 5 points, both 2- and 3-point information present together with additional information about the action of these anti-retraction systems on other bacteria and viruses.

7. Disinfection of the clinic setting was scored as: 1 point, no information available; 2 points, disinfection of the clinic settings; 3 points, information for 2-point score available together with disinfection of public areas (not including elevator); 4 points, information for 2-point score available together with disinfection of public areas (including elevator); 5 points, all information or 4-point score available together with information about virucidal cleaning products.

8. Management of medical waste was scored as: 1 point, no information available; 2 points, information about reusable instruments available (they should be pretreated, cleaned, sterilized, and properly stored); 3 points, information for 2-point score available together with information about which medical waste should be bagged conventionally; 4 points, information for 2-point score available together with information about which medical waste should be bagged with a double-layer and “goosneck” ligation; 5 points, all information for 4-point score available together with information stipulating that when suspected or confirmed 2019-nCoV infection is present, the bag should be marked and disposed according to government management.

In this way, this new evaluation tool awards a score of 8-40 points to evaluate the quality of online information relating to 2019-nCov transmission control in dental practice, considered poor (score of 8-13), medium (14-26), or high (27-40).

Statistical analysis

Data were analyzed using the SPSS version 20.0 statistical package (SPSS® Inc., Chicago, IL, USA). A descriptive study was made of each variable.

## Results

The total numbers of websites identified in Google and Yahoo! searches with content related to “2019-nCoV transmission control in dental practice” during the week 20-04-2020 to 26-04-2020, were 3,334,000 websites on Monday and Tuesday; 3,318,000 on Wednesday; 3,321,000 on Thursday; 3,319,000 on Friday; 3,329,000 on Saturday; and 3,358,000 on Sunday (Table 1).

**Table 1:**
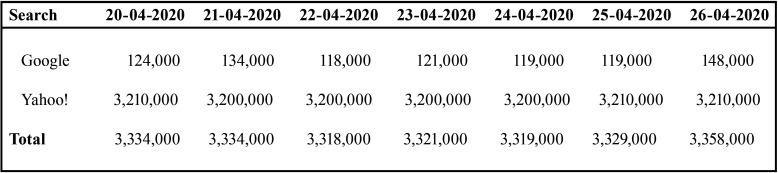
Total results of searches in Google and Yahoo! Search engines for websites relating to “2019-nCov transmission control in dental practice” during the study week (from 20-04-2020 to 26-04-2020).

On the Monday (20-04-2020), the first 100 consecutive sites in each search were visited and classified. During the following six days (21-04-2020 to 26-04-2020) only 31 additional websites were found (24 in Google and 7 in Yahoo!) within the first 100 consecutive websites identified and classified. Of the 231 websites analyzed, 201 were discarded from the study: 7 (3.50%) were duplicate sites, 12 (5.97%) were non-functioning, 41 (20.39%) were book reviews or journal abstracts, 132 (65.67%) websites failed to contain relevant information with regard to “2019-nCov transmission control in dental practice”, and 9 (4.47%) were not in English (Table 2).

**Table 2:**
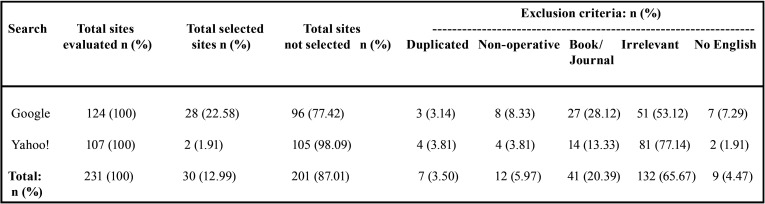
Total selected and non-selected websites identified Google and Yahoo! searches relating to “2019-nCov transmission control in dental practice” during the study week (from 20-04-2020 to 26-04-2020).

A total of 30 websites were selected for quality evaluation (Table 3). Regarding affiliation, most belonged to non-profit organizations (40%); as for specialization, 18 (60%) were websites with an area of the website (in other words, they were not exclusively) dedicated to “2019-nCoV transmission control in dental practice”; the most frequent type of information (66.67%) was based on medical facts (Table 4).

**Table 3:**
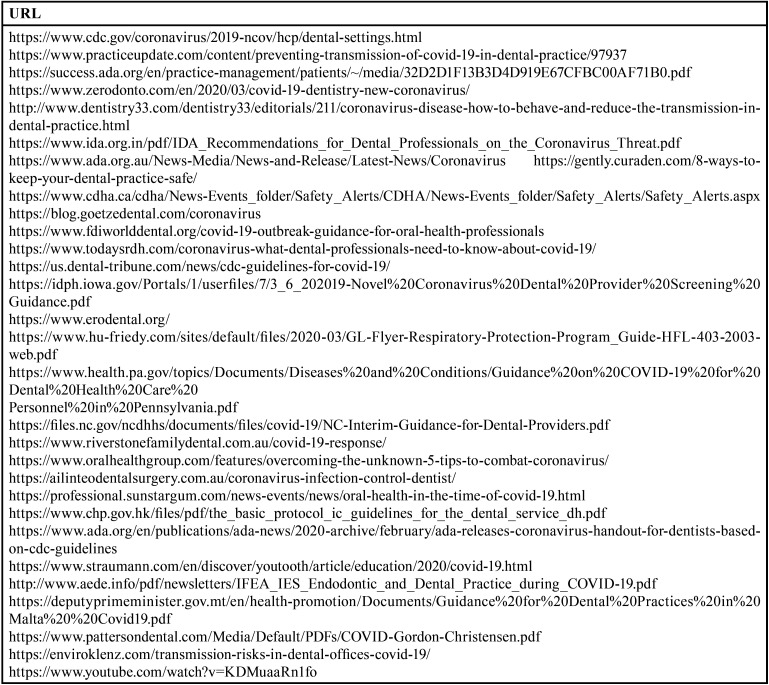
URLs of websites selected for evaluation from Google and Yahoo! searches for websites relating to “2019-nCov transmission control in dental practice” during the study week (from 20-04-2020 to 26-04-2020).

**Table 4:**
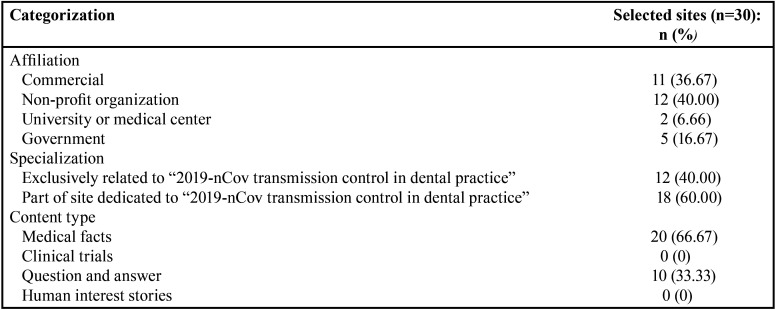
Categorization of websites based on affiliation, specialization, and type of content.

JAMA benchmark analysis found that, of the 30 websites: 3.34% did not fulfill any of the JAMA benchmark criteria, 16.67% fulfilled only 1 criterion, 30% fulfilled 2 criteria, 43.33% fulfilled 3 criteria, and 6.66% fulfilled all 4 criteria (Table 5). Regarding the HONcode, this certificate of quality did not appear on any of the websites.

**Table 5:**
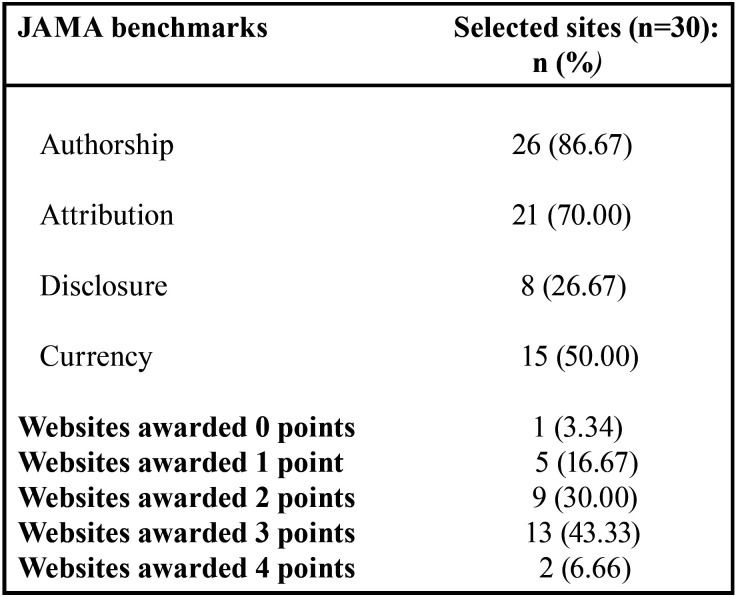
Information quality evaluation based on JAMA benchmarks.

The mean score indicating the quality of Internet websites providing information relating to 2019-nCov transmission control in dental practice was 17.63 ± 8.21 (range 8-40). The percentages of websites obtaining maximum scores (five points) for quality in each of the eight domains were: 10% for patient evaluations, 20% for hand hygiene, 10% for personal protective measures for dental professionals, 26.67% for use of mouthrinse before dental procedures, 13.33% for rubber dam isolation and high-volume suction, 16.67% for anti-retraction high-speed dental handpiece, 13.33% for disinfection of the clinic settings, and 10% for management of medical waste. Therefore, 13 (43.33%) websites were found to provide poor quality information, 14 (46.67%) provided medium quality of information, and 3 (10%) were provided high quality information (Table 6).

**Table 6:**
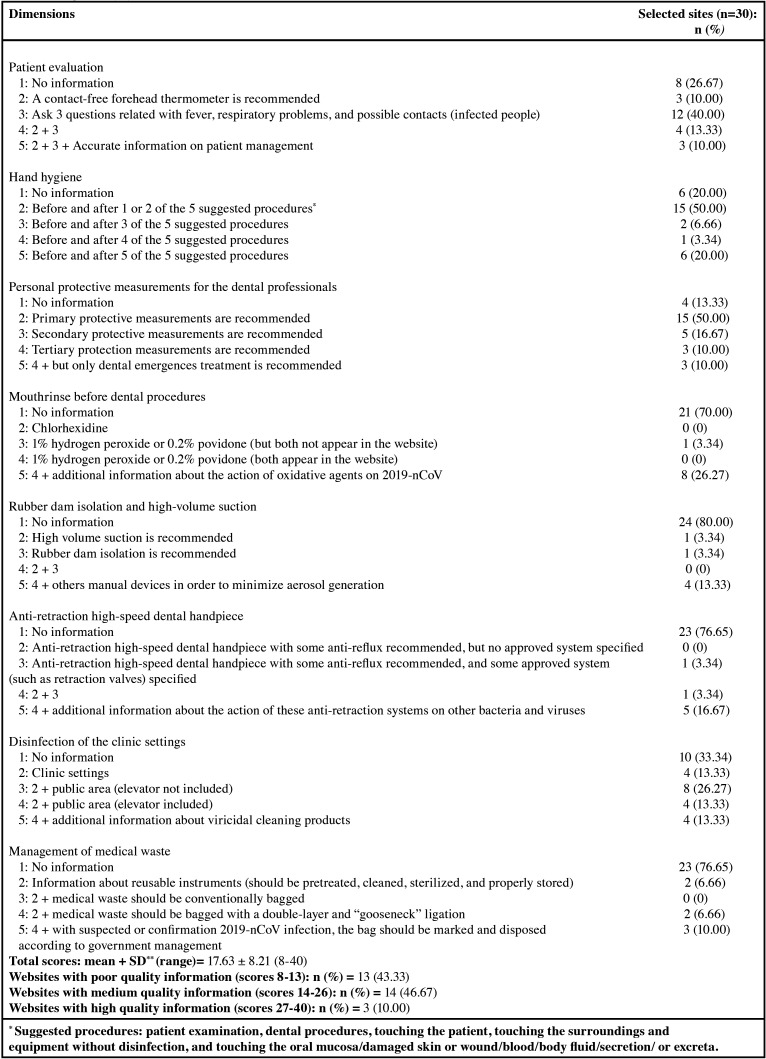
Quality of online information relating to “2019-nCov transmission control in dental practice” using a new quality of information scale based on Peng *et al.* (24).

## Discussion

In clinical practice, dental professionals (dentists, nurses, and auxiliary personnel) are exposed to a wide variety of microorganisms capable of causing diverse diseases. The mechanisms of transmission of the microbial agents can be summarized as: direct contact with lesions (saliva, blood and contaminated nasorespiratory secretions); direct contact with instruments, surfaces, contaminated dental equipment; and airborne transmission through droplet inhalation (risk increases with aerosol generation) (26) Until 2019, a declaration of principles for infection prevention and control in dental practice was in place, which was last reviewed in September 2019 and approved by the General Assembly of the World Dental Federation (FDI) (27). In addition to a set of general and specific protection measures for all dental clinical staff, the declaration includes several permanent measures against exposure such as vaccination or continuous analysis to manage possible exposure to pathogenic agents. Nevertheless, the current situation and developments since January 2020 with the appearance of 2019-nCoV or SARS-CoV-2, causing COVID-19 (6), mean that some of these measures fail to offer viable protection against this type of pathogen.

The FDI’s declaration of principles for infection prevention and control in dental practice states that information and education are fundamental factors for effectively controlling the transmission of microorganisms in dental practice. In the current worldwide situation, in which information is rarely imparted person-to-person, the Internet has become a fundamental and principal source of information (19) for dental professionals. In this context, it is very important to assess the quality of online information relating to 2019-nCov transmission control in dental practice.

In the present study, Google and Yahoo! search hits were recorded daily for one week (20-06-2020 to 26-06-2020), limiting the investigation to the first 100 websites as other researchers have done (21-23). In fact, some authors argue that according to Google ranking, the most influential websites are the first three links displayed. However, the position of a website in the first 100 search hits is not indicative of the quality of information it provides. Nevertheless, it is clear that the websites that appear after the first 100 are mostly repeats or irrelevant to the purpose of the search (21).

One of the main reasons for excluding websites from quality evaluation was the fact that many identified with the search term “2019-nCov transmission control in dental practice” proved irrelevant (65.67%), as they dealt with topics such as the control, medical and hospital management, and drug treatment of patients with COVID-19; others considered irrelevant were commercial or promotional websites, rather than educational. In fact, no standards are required of medical information on the Internet. Some websites that appear to be educational are actually promotional in nature, while others may be inefficient, incomplete, out of date, difficult to understand, or contain conflicting information (28).

Regarding the affiliation of the websites included in evaluation, only 16.67% belonged to governmental organizations, and only 6.66% belonged to Universities or medical centers. This is a relatively common finding when it comes to evaluating the quality of medical information available on the Internet, where websites mostly have commercial purposes and the information they offer may be inexact or of poor quality. For this reason, several strategies have been proposed to improve the quality of online medical information such as codes of conduct, seals guaranteeing the quality of user guides, or government regulations (29). However, during the COVID-19 pandemic it has been difficult for governments and search engines to control the quality and flow of information concerning the experiences of this pandemic (19).

As for the use of the JAMA benchmarks to measure the quality of online information, it was found that most websites (43.33%) fulfilled three criteria; however, only 6.66% of websites fulfilled all four criteria. Similarly, the study published by Cuan-Baltazar *et al.* (20), which evaluated the quality and readability of online information about COVID-19, found that only 10% of websites fulfilled all four criteria, and also that during the s months of 2020, no good quality information was available about COVID-19. As regards the HONcode, none of the websites exhibited this certification of quality, a finding that concurs with Cuan-Baltazar *et al.*, (20) who recorded the HONcode on only two websites (1.8%). But this could be due to the fact that, given the current global situation, websites have only been created very recently (28).

Applying the new tool used to evaluate the quality of internet information sites providing information about 2019-nCov transmission control in dental practice based on the study by Peng *et al.* (18), it was found that 13 (43.33%) of the 30 websites assessed provided poor quality information, 14 (46.67%) provided medium quality of information, and only 3 (10%) provided high quality information. This points to a need for better quality information about patient evaluation, hand hygiene, personal protective measurements for dental professionals, mouthrinse use before dental procedures, rubber dam isolation and high-volume suction, anti-retraction high-speed dental handpiece, disinfection of the clinic settings, and management of medical waste.

The present study suffered several limitations, the most important being the impossibility of comparing the results with any other published research, as the present work constitutes the first ever investigation analyzing the quality of online information about 2019-nCov transmission control in dental practice. Another limitation was intrinsic to the nature of the Internet, as the information available changes constantly, and as in any research, this type of paper can only analyze the information available at a particular time (30).

In conclusion, the quality of information available on websites providing information relating to 2019-nCov transmission control in dental practice is poor, which points to the need to develop strategies to improve the quality of this type of online information.
